# Supplementary material for: Single Nucleotide Polymorphisms of Toll-like Receptor 4 in Hepatocellular Carcinoma—A Single-Center Study
Source: Int J Mol Sci. 2022 Aug 21;23(16):9430. doi: 10.3390/ijms23169430 (PMC9409058; doi:10.3390/ijms23169430)
Supplement: Supplementary file 1 [file ijms-23-09430-s001.zip › ijms-1879818-supplementary.pdf]

SupplementaryMaterials

Table S1. Causes of death in patients with HCC with and without TLR4 SNPs.

Cause of death	SNPs	No SNPs	p value*
**Liver-related**	5	27	>0.999
Liver failure	2	16	0.678
Spontaneous Bacterial Peritonitis	1	4	>0.999
Variceal bleeding	2	4	0.257
Hepatorenal syndrome	1	3	0.532
**Non liver-related**	2	8	>0.999
Sepsis	2	6	0.601
Heart failure	0	2	>0.999

Abbreviations: *SNPs: single nucleotide polymorphisms*

*p values are based on Fischer's exact test

Table S2: Characteristics of patients with HCC in relation to the presence of TLR4 SNPs.

Characteristic	SNPs (N=10)	no SNPs (N=42)	p-value*
Age in years, median (Q1-Q3)	59.5 (55-72)	69.5 (63-74)	0.270
Gender (males), N (%)	9 (90%)	33 (78.6%)	0.664
Smoking, N (%)	2 (20%)	10 (23.8%)	>0.999
Alcohol (Heavy drinkers), N (%)	3 (30%)	15 (35.7%)	>0.999
Hgb, median (Q1-Q3)	11.15 (10-11.8)	10.75 (9.1-12.6)	0.530
PLTs, median (Q1-Q3)	112 (97-154)	138.5 (91-188)	0.353
AST(IU/ml), median (Q1-Q3)	59.5 (37-71)	52 (36-104)	0.944
ALT(IU/ml), median (Q1-Q3)	31 (27-55)	39 (20-70)	0.981
gGT(IU/ml), median (Q1-Q3)	156 (112-245)	86.5 (44-216)	0.157
ALP(IU/ml), median (Q1-Q3)	127 (116-157)	157 (91-235)	0.693
Total bilirubin (mg/dl), median (Q1-Q3)	1.76 (0.88-2.52)	1.285 (0.9-3.15)	0.963
INR, median (Q1-Q3)	1.3 (1.14-1.63)	1.34 (1.18-1.84)	0.754
Urea, median (Q1-Q3)	39 (25-47)	42.5 (34-79)	0.312
Creatinine, median (Q1-Q3)	0.82 (0.7-1)	0.975 (0.72-1.45)	0.408
Cholesterol	156 (110-200)	133 (101-172)	0.378
MELD score, median (Q1-Q3)	12 (9-18)	11 (10-18)	0.898
CTP score, median (Q1-Q3)	7 (6-11)	7.5 (6-9)	0.972

Abbreviations: *ALT: Alanine aminotransferase; ALP: Alkaline phosphatase; AST: Aspartate aminotransferase; CTP: Child-Turcotte-Pugh score; gGT: Gamma-glutamyl transferase; Hgb: Hemoglobin; INR: International normalized ratio; MELD: Model for end-stage liver disease; PLTs: Platelets; SNPs: Single nucleotide polymorphisms*

* p values represent Fischer exact test for categorical data and Mann Whitney U test for the numerical data
